# Individualized 3D printed navigation template-assisted atlantoaxial pedicle screws vs. free-hand screws for the treatment of upper cervical fractures

**DOI:** 10.3389/fsurg.2022.932296

**Published:** 2022-09-26

**Authors:** Guoqi Niu, Jiawei Cheng, Lutan Liu, Chao Li, Gong Zhou, Hui Chen, Tao Liu, Hu Nie, Zheng Sun, Weili Jiang, Qiankun Zhou, Baoyin Zhao, Jun Zhu, Ruochen Yu, Yalong Guo, Yi Yang, Jianzhong Bai

**Affiliations:** ^1^ Department of Orthopedics, The Second Affiliated Hospital of Bengbu Medical College, Bengbu, China; ^2^ Digital Orthopedics Technology R/D and Application Innovation Team

**Keywords:** upper cervical deformity, 3D printing, guide template, pedicle screw, upper cervical fractures

## Abstract

**Objective:**

This study aims to compare the efficacy and safety of freehand atlantoaxial pedicle screws against custom 3D printed navigation template screws in the treatment of upper cervical fractures.

**Methods:**

In our institution from 2010 to 2020, a retrospective cohort analysis of 23 patients with upper cervical fractures was done. These patients were separated into two groups: group A (*N* = 12), which received customized 3D printed navigation template-assisted screws with virtual reality techniques, and group B (*N* = 11), which received freehand screws assisted by intraoperative fluoroscopy. Every patient was monitored for more than 1 year. The two groups were contrasted in terms of screw implant accuracy, cervical spine Japanese Orthopaedic Association (JOA) score, American Spinal Injury Association (ASIA) score, visual analogue scale (VAS) score, surgical time, fluoroscopy times, and intraoperative blood loss.

**Results:**

A total of 88 atlantoaxial pedicle screws in all, 46 in group A and 42 in group B, were implanted. In group A, the screw insertion accuracy rate was 95.7%, compared to 80.0% in group B (*P *< 0.05). When compared to group B, group A had shorter surgery times, less blood loss, fewer fluoroscopies, a higher short-term JOA score, and overt pain reduction (*P *< 0.05). However, there was no discernible difference between the two groups' VAS scores, long-term JOA scores, or ASIA scores (sensory and motor), at the most recent follow-up.

**Conclusion:**

Individualized 3D printed guide leads to significant improvement in the screw safety, efficacy, and accuracy, which may be a promising strategy for the treatment of upper cervical fractures.

## Introduction

The upper cervical vertebra, which includes the atlas and axis, is located at the transition area of craniocervical function and adjacent to important anatomical structures, such as the vertebral artery, medulla oblongata, and cerebellum (1). As a result, the structural destruction of the upper cervical vertebra is likely to cause damage to the spinal cord or vertebral arteries, which could result in paralysis, respiratory failure, and a reduction in cardiovascular activity, all of which could significantly raise the morbidity and mortality of patients. Trauma is the main reason for upper cervical spine fractures (2). However, there is not much research on upper cervical spine fractures, owing to the fact that most patients with atlas or axis fractures with atlantoaxial joint dislocation, spinal cord injury, or vertebral artery injury passed away at the scene of the accident.

The pedicle of the upper cervical vertebra exhibits a high degree of anatomic variability and asymmetry. There is no vertebral body and no lamina structure because of the unique architecture of the atlas, and hence no pedicle in the traditional sense of the word. Although it shares anatomical and functional similarities with the pedicles of other cervical vertebrae, the link between the posterior arch of the atlas and the lateral mass is referred to as the pedicle of the atlas to aid in clinical understanding (3). According to several studies, 20% of the atlas pedicles contain anatomical variance (4, 5). It should be noted that the pedicle for the axis has a limited diameter, measuring one-fifth less than 3.5 mm (6). The rate of fluctuation in the vertebral artery at the atlantoaxial joint is also higher (7). As a result, even skilled spine surgeons have trouble precisely inserting screws into the atlas or axis pedicles. Once the implant location has changed, it may leave the patient disabled or even put their lives in danger (8).

The main course of treatment for fractures of the upper cervical spine is posterior pedicle screw fixation. Its primary goals are to relieve spinal cord and nerve compression and to restore the normal height of the intervertebral space, the physiological curvature of the cervical spine, and the stability of the cervical spine. However, the outcome of the surgery depends on how accurately the screws are implanted. Only 75% of screws placed by hand accurately in the atlantoaxial pedicle have been documented (9). Additionally, the operation takes a long time to complete, results in significant blood loss, necessitates numerous fluoroscopy inspections throughout the surgical procedures, and is challenging to do with accurate screw placement, all of which lower the operation's overall success rate.

In recent years, 3D printing technology has advanced rapidly, and it is now extensively employed in many fields, particularly orthopedics (10–12), for difficult hip and knee replacements, pelvic fractures, pilon fractures, tumor-induced bone deformities, etc. The effectiveness, precision, and success rate of the procedure are all considerably increased by this technology. In order to increase the precision and safety of screw placement, this study used customized 3D printed navigation templates to assist in the implantation of atlantoaxial pedicle screws.

## Materials and methods

In our institution between 2010 and 2020, a retrospective cohort analysis was performed on 23 patients who had upper cervical fractures. These patients were split into two groups: those who received customized 3D printed navigation templates helped screws with virtual reality techniques (group A, *N* = 12), and those who received freehand screws assisted by intraoperative fluoroscopy (group B, *N* = 11). A total of 46 and 42 atlantoaxial pedicle screws were used in group A and group B, respectively. Using the Kawaguchi method, the screw placement's accuracy was evaluated. Every patient was monitored for more than a year. Screw implant accuracy, cervical spine Japanese Orthopaedic Association (JOA) score, American Spinal Injury Association (ASIA) score, visual analog scale (VAS), surgery time, fluoroscopy times, and intraoperative blood loss were all compared between the two groups. This type of surgical procedure has been approved by our hospital's ethics committee, and all patients have completed the consent form.

### Selection criteria

Inclusion criteria are as follows: (1) upper cervical fractures are diagnosed; (2) the patient is older than 18 years old; (3) their physical condition must be good, and there must be no overt medical reasons why surgery shouldn't be performed.

Exclusion criteria include the following: (1) the amount of pedicle variability is too great to be corrected by pedicle screws; (2) the underlying disease cannot be operated on; (3) the follow-up data are insufficient.

### Orientation template design

Prior to surgery, patients in group A got a spiral CT scan (Siemens, Germany). The upper cervical spine's CT data were transmitted to Mimics 17.0 (Materialise, Belgium) for modeling after being converted into DICOM format for storage. To avoid any virtual screws penetrating the pedicle's four walls, choose the target vertebral body in the Mimics virtual software, extract the atlantoaxial pedicle's anatomical structure from the back of the deformed vertebral body, create a virtual pedicle screw, and fit the best pedicle screw entry point, the screw channel, and the length of the screw. Create a guide tube using the software with the following dimensions: 0.4 mm for the inner diameter, 8 mm for the outer diameter, and 15–20 mm for the length. The final individualized atlantoaxial pedicle screw placement is fitted through the “Boolean calculation” in the software orientation template, taking into account the anatomical shape of the back of the atlantoaxial vertebral body. We designed the template according to the behind anatomy of the atlas and axis using the mirroring and reverse forming techniques. The template can be directly stuck on the atlas or the axis.

### Model, guide plate printing

First, check the fidelity of the model printing by importing the saved STL data into ideaMaker (Jiangsu, China). The upper cervical spine model should next be saved in GCODE format, imported into a 3D printer (Shanghai Maiditu Company), and printed at a 1:1 scale using PLA as the printing material. The model base, internal and external support, and upper cervical spine model were printed using the following printing parameters: single layer height of 0.25 mm, filling rate of 10.0%, and printing speed of 70.0 mm/s. Additionally, print off 10 upper cervical spine models and 40 guidance templates, then practice your surgery on them. A 1.5 mm Kirschner wire is used by the surgeon to make holes under the sleeve's shield. Watch the Kirschner wires' route in the model's pedicle if they are drilled to the same length as the screw generated by the preoperative program in such situation. If the Kirschner wires are placed in the pedicle, a guide template is thought to be helpful. At this moment, the intraoperative screws are chosen based on the depth of the Kirschner wires in the pedicle.

### Surgical approach

#### Group A

**Figure 1:**
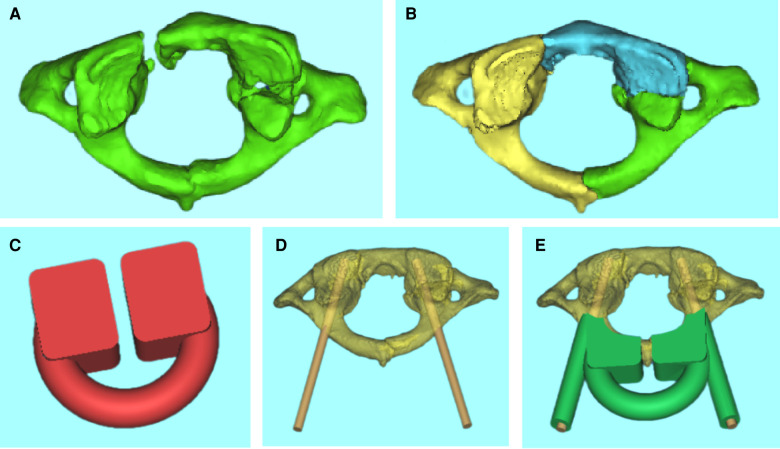
(**A–E**) Virtual reduction of the fracture fragments and individualized 3D printed navigation template were prepared according to the angle of screw placement.

**Figure 2:**
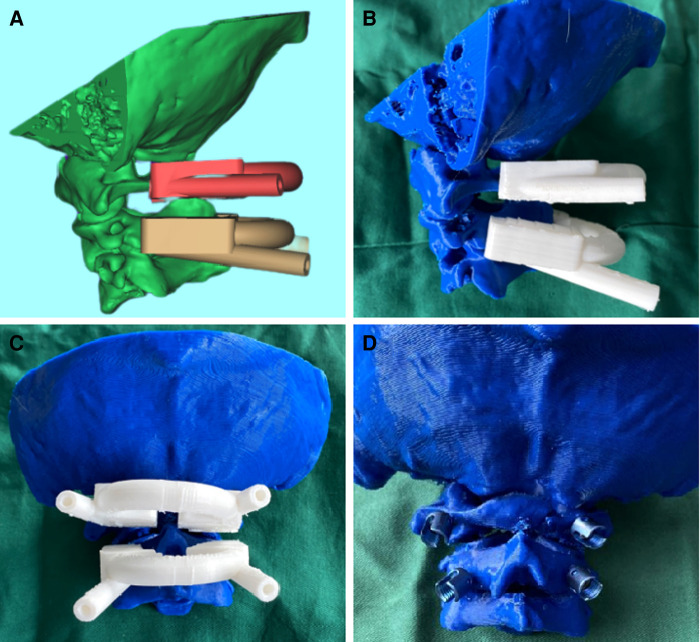
(**A–D**) 1:1 printing of the vertebral body model and preoperative simulation.

**Figure 3:**
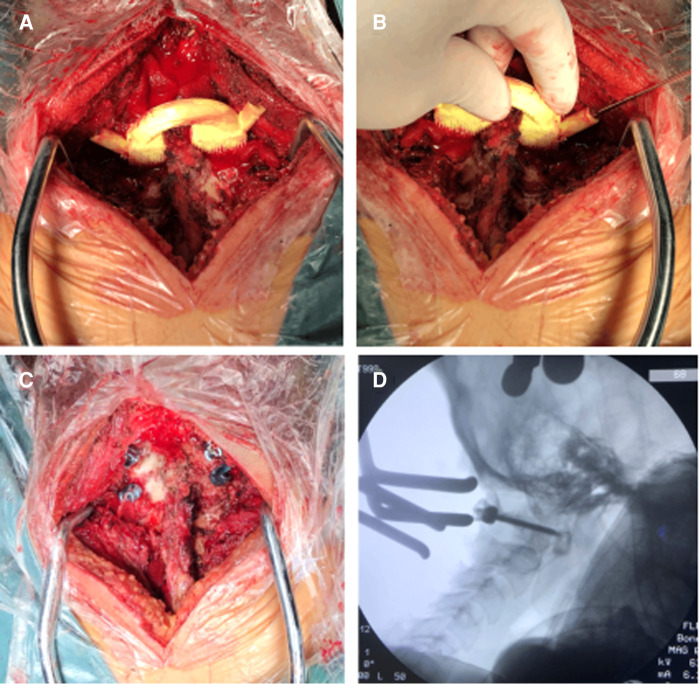
Operation process: (**A**) the matching degree of the guide plate was tested again before screwing; (**B**) the corresponding segment guide template was attached after exposure of atlantoaxial vertebrae; (**C**) the hole was drilled with Kirschner wire under the protective sleeve, and tap after the four walls are complete; (**D**) intraoperative fluoroscopy was used to verify the accuracy of screw placement.

Individualized 3D printed navigation templates were created in accordance with the angle of screw placement using the software to perform a virtual reduction of the fracture fragments (Figures 1A–E). Next, 1:1 printing of the vertebral body model and preoperative simulation were performed (shown in Figures 2A–D). First, the patient underwent general anesthesia and was positioned supine, with the neck hyperextended, and 3 kg of treatment weight. To expose the C_1_ posterior arch and C_2_ lamina, make a median longitudinal incision behind the neck while the patient is lying on his or her back. Then, using the periosteal device, separate the posterior atlantoaxial muscles and suboccipital muscles for periosteal dissection. We exposed the bony structure of the atlantoaxial and then clipped the template to the atlas or the axis. While the K-wire is being performed, the template is held in place by hand (Figure 3A). The surgeon drills through the sleeve while using a 1.5 mm needle, measuring the depth of the hole in proportion to the lengths that were recorded before and after the simulation procedure (Figure 3B). Once the guide plate has been taken off, the probe is used to check the pedicle's four walls to make sure the screw path is completely inside the cortex. Once the location is good from the perspective, the tap is gradually adjusted, the depth is measured again, the right length of the screw is chosen to slowly screw in, and the screw is inserted. The screw is inserted into the axis using the same procedure (Figure 3C). Perform intraoperative fluoroscopy following the insertion of the screw (Figure 3D). Once the screws have been entirely implanted, two rods were fixed.

#### Group B

The surgical approach is similar to that of group A, with the exception that the surgeon implants the screw by hand based on the imaging evaluation and prior experience.

#### Postoperative treatment

(1) Cervical collar underwent after fixation, keeping the neck in a neutral position as much as possible to prevent overextending through flexion; (2) preoperative dexamethasone and intravenous omeprazole in 2–3 days, preventing surgery-induced spinal cord edema and stress ulcers brought on by the operation; conventional anti-infection, nutritional nerve, analgesic drugs, and other supportive treatment; (3) anterior surgery to strengthen oral care, surgical incisions are regularly cleaned and dressing changed, beware of oropharynx and infection of the incision at the back of the neck; (4) drainage is frequently implanted, and the drainage volume is less than 50 ml within 24 h to remove, to minimize prolonged installation and increased infection risk.

#### Outcome evaluation index

**Figure 4:**
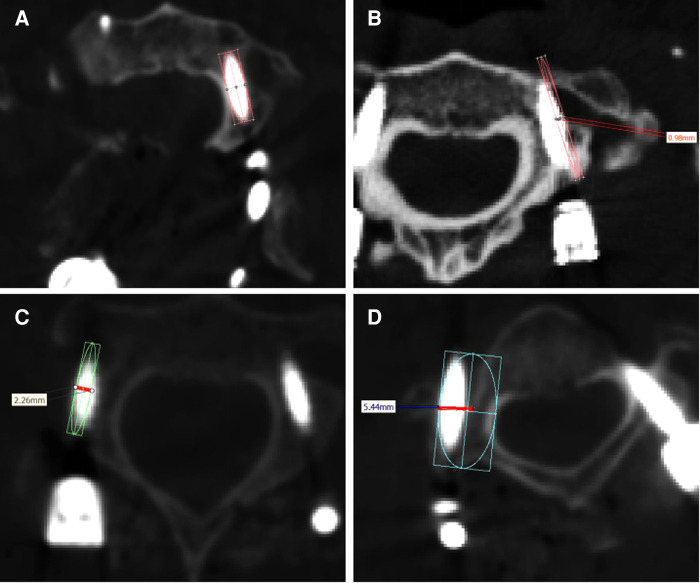
(**A–D**) Evaluation criteria for screw implantation quality.

Following surgery, all patients underwent a three-dimensional computed tomography scan of the cervical region, and the Kawaguchi method was used to assess the precision of screw placement based on the findings of the postoperative CT imaging (13). Grade 0 screws are those that do not pierce the pedicle (Figure 4A), grade 1 screws are those that do so without causing complications (Figure 4B), grade 2 screws are those that do so without causing complications (Figure 4C), and grade 3 screws are those that cause related complications (Figure 4D). Grade 0 and 1 screws were identified in this investigation as being of high quality. The VAS, the ASIA score, and the JOA score are often used as evaluation indicators for patients after cervical spine surgery.

### Statistical analysis

The data processing program utilized was Graphpad Prism 9. The *t*-test was used to examine data that were measured, as well as the chi-square test. *P*-values less than 0.05 were considered statistically significant.

## Results

### Characteristics of patients

The average age of the 12 patients in group A was 47.8 years; there were nine men and three women in total. Injuries that are related to motor vehicle accidents account for 50%, falls from great heights account for 25%, and falls on the ground account for 25%. All of the patients experienced neck pain, including five who had difficulty swallowing and nine who reported paresthesia or muscle paralysis. Nine men and two women made up group B, and their average age was 42.5 years. Traffic accidents (45.5%), falls from great heights (9%), and falls on the ground are the main causes of injuries (45.5%). All patients experienced neck pain, including four who had dysphagia and eight who had paresthesia or muscle weakness. There were no significant differences between the two groups in terms of the general data of the included patients (shown in Table 1).

**Table 1:** Baseline characteristics of patients.

	Group A	Group B
Age (year)	47.8 ± 11.4	42.5 ± 16.7
Gender, no (%)		
Female	3 (25)	2 (18)
Male	9 (75)	9 (82)
Cause of injury, no (%)		
Traffic accident	6 (50)	5 (45.5)
Fall from height	3 (25)	1 (9)
Fall on the ground	3 (25)	5 (45.5)
Symptoms, no (%)		
Neck pain	12 (100)	11 (100)
Dysphagia	5 (42)	4 (36)
Paresthesia/weakness	9 (75)	8 (73)

### Clinical outcomes

**Figure 5:**
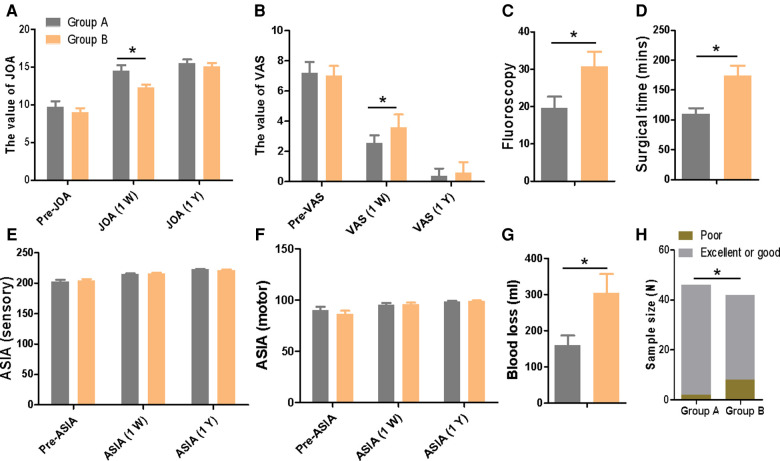
(**A–G**) Comparison of operation time, blood loss, fluoroscopy times, excellent and good rate, VAS, ASIA, JOA, Blood loss, and the rate of excellent or good between the two groups.

**Figure 6:**
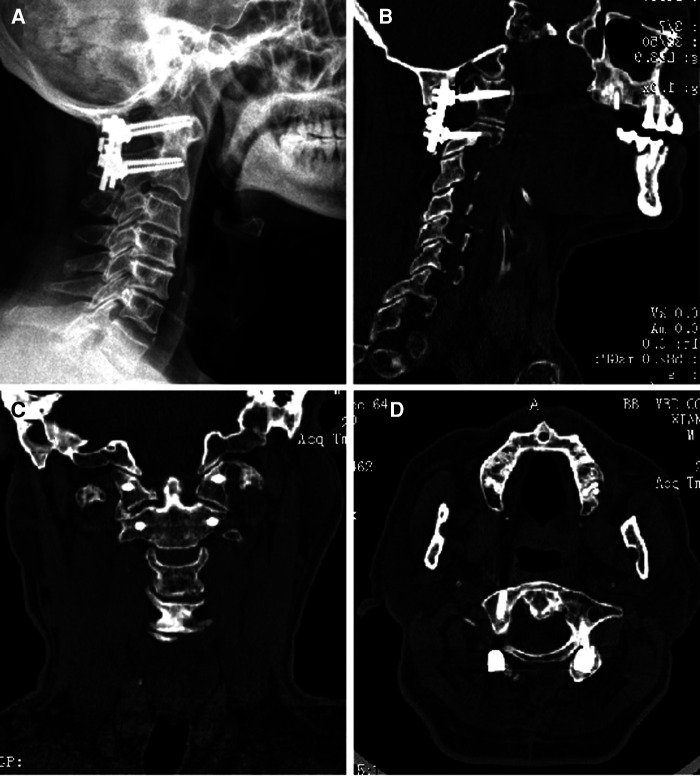
Postoperative x-ray and CT imaging data.

The study comprised a total of 23 patients with upper cervical fractures. A total of 88 atlantoaxial pedicle screws (46 in group A and 42 in group B) were implanted. The accuracy rate of group A for screw implantation was 95.7% and that of group B was 80.0%, with *P *< 0.05 (shown in Figure 5H). The surgery took less time for group A (110.0 ± 31.9 min) than for group B (173.8 ± 53.3 min), with *P* < 0.05 (Figure 5D). Regarding intraoperative blood loss, there was a significant difference between group A (159.6 ± 90.1) and group B (304.0 ± 167.5), with *P* < 0.05 (Figure 5G). The intraoperative fluoroscopic times in groups A and B were 17.2 ± 8.4 and 30.7 ± 12.7, respectively (*P* < 0.05) (Figure 5C). One week after surgery, group A's VAS scores (2.6 ± 0.5) were lower than group B's (3.6 ± 0.8) (*P* < 0.05) (Figure 5B). One week after surgery, group A's JOA score was considerably greater than that of group B's (*P* 0.05) (Figure 5A). In contrast, there was no difference between the two groups' VAS ratings, last follow-up JOA scores, or ASIA scores (*P* > 0.05) (Figures 5A,B,E,F). The screws are in the pedicle, according to postoperative x-ray and CT imaging data (Figure 6).

### Postoperative complications

Postoperative problems were not experienced by any of group A patients. Two patients (18%) in group B experienced postoperative problems. Following surgery, one patient experienced a fever, which was treated with antibiotics and dressing changes by returning to normalcy. The other patient experienced cerebrospinal fluid leakage, but the symptoms disappeared after intensive dressing changes.

## Discussion

The cervical spine's rotation and flexion are performed by the atlantoaxial joint, which has a relatively large range of motion but lacks stability due to its unique architecture (14). The fracture of the atlantoaxial joint is more likely to cause instability, which will disrupt the alignment of the joint and lead to atlantoaxial dislocation (14, 15). The compression of the spinal cord may reduce the muscle strength of the limbs due to variations in these anatomical features. In extreme circumstances, respiratory depression may result in paralysis or even death. Conservative methods of treatment are useless, and prolonged conservative therapy may even stall the progression of the illness, leaving the patient in excruciating discomfort. Therefore, it is crucial to find a suitable and efficient treatment strategy as soon as a patient is clinically determined to have an upper cervical spine fracture. The active surgical intervention in the upper cervical spine sequence is used to maintain the proper anatomical relationship of the vertebral body, relieve compression on the tissues surrounding the upper cervical spine, and correct and maintain the unstable spinal sequence.

After atlantoaxial reduction, the cervical spine posterior internal fixation method has proven to be the most efficient way to continue maintaining the correct anatomical relationship of the vertebral body. Gallie first suggested using wire binding in conjunction with autogenous iliac bone graft fusion, but this surgical approach has a number of drawbacks (16), including subpar internal fixation biomechanical stability, spinal cord injury from wires, and a high rate of postoperative sequelae. Pedicle screw technology has gradually taken over as the primary technique for posterior cervical internal fixation due to the medical device industry's rapid development (17). However, a more advanced level of pedicle fixation technology is necessary because of the unique anatomical anatomy of the pedicle of the atlantoaxial vertebra itself. Because of its great stability, high rate of fusion, and effective position correction, posterior atlantoaxial pedicle screw fixation is widely acknowledged by spine surgeons (18, 19).

The two-dimensional planar imaging data of the patient taken before the procedure and the pertinent anatomical landmarks used during the procedure are largely what the traditional freehand approach of screw insertion depends on. The fact that this approach does not require expensive or sophisticated equipment to help, even though low-level hospitals have established technology that can also be employed, is a big advantage. The steep learning curve, meticulous execution required throughout the procedure, high standards for the surgeon, and excessive reliance on the surgeon's prior experience make it impossible to ensure screw placement accuracy using this method. The amount of radiation exposure for the patient and the surgical team is also increased by the necessity of a significant number of x-ray fluoroscopy aids during the procedure (20). Furthermore, it takes a long time for the freehand screw placement technique to mature and is very subjective. The three-dimensional CT imaging data of the patient's upper cervical spine is obtained during the procedure and imported into a virtual program that is part of the computer navigation system for real-time reconstruction. Under computer guidance, sensitive tissues like nerves and blood vessels are avoided, the best insertion point is chosen, and the screw insertion is then finished once the angle has been adjusted. Computer technology was employed by Richter et al. to assist with cervical pedicle screw placement, and the results showed that just 3% of navigation screws were placed incorrectly (21). According to Shin et al., the use of navigation technology can lessen the likelihood that a screw will penetrate the pedicle and lessen the nerve and blood vessel damage that a screw placement might cause (22). Computer-assisted screw insertion does have certain drawbacks, though, including the price, complexity, and steep learning curve of the equipment. Additionally, the position of the patient cannot be altered throughout the procedure; otherwise, it must be adjusted. Prior to surgery, 3D printing technology is used to acquire three-dimensional CT data of the patient's upper cervical spine, rebuild it using computer software, and then print a 1:1 replica of the model. A guiding template for screw placement was created to aid in the accurate and speedy positioning of the screw during the surgical process based on the anatomical features of the upper cervical spine. Moreover, the printing of the template will not delay the patient's surgery. First of all, the patients with upper cervical spine fractures we included were all elective surgery, not emergency surgery. In addition, after obtaining the patient's CT examination, we could complete the printing and sterilization of the template within 12 h.

### Experience with upper cervical spine surgery

(1) The interspinous and supraspinous ligaments are preserved during surgery because the guide plate is built as an arch bridge structure, and the guide sleeves on either side are joined by an arc structure. (2) To ensure that the customized 3D printed navigation template closely fits the bone surface, the soft tissue at the screw placement location should be eliminated as much as possible. (3) To avoid drilling through the anterior edge of the vertebral body, the screw length should be precisely measured in the 3D printed model. (4) To reduce friction, paraffin oil is dripped into the guide sleeve during the procedure. The guide sleeve should have a slightly larger diameter than the K-wire. (5) To avoid deformation brought on by high temperature, it is advised to disinfect the guide plate using ethylene oxide or low-temperature plasma. (6) The height of the medial one-third of the posterior arch of the atlas is smaller than the lateral one-third, so it is safer to place the screw in the lateral one-third of the posterior arch below the vertebral artery groove. When the height of the lateral one-third of the posterior arch is less than 3.5 mm, pedicle screws are generally not used because the vertebral artery may be damaged. Lateral mass screws are optional.

## Conclusion

The individualized 3D printed guide leads to significant improvement in screw safety, efficacy, and accuracy, which may be a promising strategy for the treatment of upper cervical fractures.

## Data Availability

The raw data supporting the conclusions of this article will be made available by the authors, without undue reservation.
